# Findings from Community-Based Screenings for Type 2 Diabetes Mellitus in at Risk Communities in Cape Town, South Africa: A Pilot Study

**DOI:** 10.3390/ijerph17082876

**Published:** 2020-04-21

**Authors:** Jillian Hill, Nasheeta Peer, Deborah Jonathan, Mary Mayige, Eugene Sobngwi, Andre Pascal Kengne

**Affiliations:** 1 Non-Communicable Diseases Research Unit, South African Medical Research Council (SAMRC), Cape Town 7505, South Africa; nasheeta.peer@mrc.ac.za (N.P.); Deborah.Jonathan@mrc.ac.za (D.J.); andre.kengne@mrc.ac.za (A.P.K.); 2 National Institute for Medical Research, Dar es Salaam 11000, Tanzania; maryma13@yahoo.com; 3 Department of Medicine, University of Yaounde, Yaounde 00000, Cameroon; sobngwieugene@yahoo.fr

**Keywords:** diabetes risk, community screening, cardiovascular disease, hypertension, dyslipidemia

## Abstract

Completed and ongoing implementation activities globally advocate for community-based approaches to improve strategies for type 2 diabetes prevention. However, little is known about such strategies in the African region where there are higher relative increases in diabetes prevalence. We reported findings from the first 8-month pilot phase of the South African diabetes prevention program. The study was conducted across eight townships (four black and four mixed-ancestry communities) in Cape Town, South Africa, between August 2017 and March 2018. Participants were recruited using both random and self-selected sampling techniques because the former approach proved to be ineffective; <10% of randomly selected individuals consented to participate. Non-laboratory-based diabetes risk screening, using the African diabetes risk score, and based on targeted population specific cut-offs, identified potentially high-risk adults in the community. This was followed by an oral glucose tolerance test (OGTT) to confirm prevalent pre-diabetes. Among the 853 adults without prior diabetes who were screened in the community, 354 (43.4%) were classified as high risk, and 316 presented for further screening. On OGTT, 13.1% had dysglycemia, including 10% with screen-detected diabetes and 67.9% with glycated haemoglobin (HbA1c)-defined high risk. Participants with pre-diabetes (*n* = 208) had high levels of common cardiovascular risk factors, i.e., obesity (73.7%), elevated total cholesterol (51.9%), and hypertension (29.4%). Self-referral is likely an efficient method for selecting participants for community-based diabetes risk screening in Africa. Post-screening management of individuals with pre-diabetes must include attention to co-morbid cardiovascular risk factors.

## 1. Background

Increasingly, community-based risk screening actions are advocated to reduce the growing global burden of type 2 diabetes mellitus (T2DM). The burden of T2DM in sub-Saharan Africa (SSA) is substantial and growing rapidly. The population of people with diabetes in SSA is expected to increase by 142.9% from 19.4 million in 2019 to 47.1 million people in 2045 [1]. Of the countries in SSA, South Africa already has the second largest number of people with diabetes. In 2017, there were 1,826,100 cases of diabetes recorded in adults [2], with the greatest burden experienced by socio-economically disadvantaged populations. Recent prevalence rates in these populations, i.e., 13.1% in blacks [3] and 26.3% in mixed ancestry [4], are similar to those in populations known to be at high-risk for T2DM. While appropriate management of people already diagnosed with T2DM is essential, identifying those at high-risk and preventing or postponing the progression to T2DM is equally important.

Although there is definitive evidence on the effectiveness of lifestyle interventions in preventing diabetes among high-risk individuals [5,6], little is known about implementing these interventions in real-life settings in SSA. The South African diabetes prevention program (SA-DPP) aims to develop and evaluate a culturally relevant T2DM prevention model for South Africa, using evidence from successful diabetes prevention effectiveness and implementation programs [7,8,9,10]. The goal is to arrive at a model that will inform lifestyle interventions to prevent T2DM and other lifestyle-related conditions at primary health care level in South Africa. This may serve as a model for adaptation in other SSA countries that are confronted with similar challenges as South Africa. The first phase of this study was the recruitment and screening process. In this manuscript, we reported on the findings of the screening activities of the SA-DPP pilot study. 

## 2. Materials and Methods

### 2.1. Study Design and Population

The target population included black and mixed-ancestry participants between the ages of 25 and 65, without known diabetes. The SA-DPP sample size was calculated, assuming a cumulative incident diabetes rate of 13.6% at 2–3 years, as observed in our Bellville South cohort [11], with an expected relative risk to be 0.51, which is the pooled effect estimate of efficacy trials comparing lifestyle intervention vs. usual care in preventing [12]. We further assumed an intra-cluster correlation coefficient (ICC) for fasting glucose of 0.02 [13]. The sample size calculation was based on the above at a significance level of 5% with a type II error risk of 20% and with an estimated 36 months loss to follow-up of 20–25%. Recruiting 56 clusters of 20 participants would provide adequate power for detecting a significant effect on incident T2DM after three years of follow-up in those with pre-diabetes. Importantly, the study would be able to demonstrate significant changes in intermediate outcomes (weight and body mass index (BMI)) after the first year of the intervention. This, in turn, would inform the scalability of the project ahead of completion.

The pilot sample of the SA-DPP comprised eight low-socioeconomic townships in Cape Town, South Africa, with four groups each from the black and mixed-ancestry communities (Table 1). This would enable equal distribution of the intervention and control arms by population groups. The pilot study thus consisted of 160 participants (eight groups ×20 participants), which was about 14% of the total sample required for the intervention phase. To identify 160 individuals at high-risk for T2DM, it was estimated that 910 individuals would need to be invited to undergo screening. With an estimated response rate of 80%, it was expected that 728 individuals would avail themselves for home screening. Of these, 364 would require oral glucose tolerance tests (OGTTs), and 160 were expected to be identified as high-risk. 

### 2.2. Participants Selection

The details of the included eight townships (four black and four mixed ancestry) are included in Table 1. Participants were initially selected using a random sampling technique. However, this did not prove successful, and the self-selection approach was adopted for pragmatic reasons. In the random selection approach, aerial maps of the included townships (obtained from the municipality of Cape Town) were used to randomly select households. GPS coordinates were randomly selected, and address lists were generated. Fieldworkers then visited these households to identify and screen potentially eligible participants. Participants were eligible for participation if they were between the ages of 25–65 years, without known diabetes. People were excluded if they were bedridden, pregnant, or breastfeeding, or were in receipt of cancer or tuberculosis treatment within the past three months. Households were visited at least three times before generating new lists. This method proved to be unsuccessful in the first four areas, with less than a 10% response rate. Reasons included people not being at home or simply not wanting to open the door to strangers (with safety being a real issue in these communities), or not being eligible to participate, i.e., not fulfilling the inclusion criteria. In the self-selection approach, the study was advertised through local councilors’ offices, churches, schools, as well as flyers, being distributed door-to-door (or dropped in post-boxes) in the community. Potentially interested participants could then come to pre-determined venues in their community for community-based risk screening.

### 2.3. Diabetes Risk Screening

Diabetes risk screening followed a two-stage approach: a community-based risk screening using a risk questionnaire, followed by a clinic-based risk status assessment using biochemical analyses.

### 2.4. Community-Based Diabetes Risk Screening

**Figure 1:**
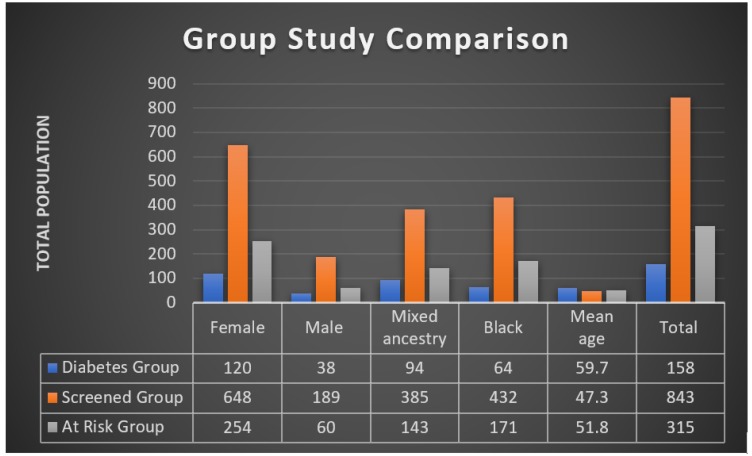
Community-based screening group comparison.

The development and use of diabetes risk scores based on self-reported or clinical data for both the detection of T2DM and the identification of individuals at high-risk for future T2DM have become evident in recent years. However, the application to low- and middle- income countries, such as those in SSA, remain unclear [18]. Mayige (2014) [19] derived and validated a simple risk score for undiagnosed T2DM in African populations. Age, hypertension, and waist circumference were included in the final model of the African diabetes risk score (ADRS) [19]. The SA-DPP made use of ADRS for community-based T2DM risk screening. Local data and receiver-operating characteristics (ROC) analyses were used to optimize ADRS thresholds for black [3] and mixed-ancestry [4] South Africans, which were 1.46 and 1.15%, respectively (Figure 1). 

Trained fieldworkers conducted community-based risk screening by administering the brief screening questionnaire (age, gender, and ethnicity) and measuring anthropometry and blood pressure (BP). Three BP measurements were taken at two-minute intervals using an Omron BP monitor after the participant had been seated for five minutes. Height, weight, and waist measurements were measured using standardized techniques [20]. This was used to estimate the risk of T2DM by the ADRS (see parameters in Table 2). Participants deemed at high-risk were referred to our research clinic for biochemical investigations. Participants with blood pressure levels considered to constitute an immediate risk for their health (systolic blood pressure (SBP) ≥ 140 and or diastolic blood pressure (DBP) ≥ 90 mmHg) were referred to a nearby public health facility for further management. 

### 2.5. Clinic-Based Diabetes Risk Confirmation and Baseline Measurements

Participants deemed at high-risk during community-based screening were transported on pre-determined dates to our research clinic for further assessment by our dedicated research drivers. Baseline assessments included OGTTs and other biochemical and clinical assessments. Blood samples for glucose and lipids were drawn after a 10-hour overnight fast, then a standard OGTT using 75 g of anhydrous glucose in 250 mL of water was administered, and blood samples were taken 120 min later [21]. A qualified nurse collected the blood samples. 

The trained fieldworkers with ≥ 1 year of experience administered the questionnaire and performed the anthropometric and BP assessments. Among the data collected were the following: socio-demographic information, personal and family medical history, dietary data using a single non-quantified 24-hour recall, physical activity (global physical activity questionnaire (GPAQ)) [22], and physical environment (neighborhood environment walkability scale (NEWS) Africa) [23] (Table 3). Anthropometric and BP measurements were repeated using the standardized techniques described above.

### 2.6. Data Entering and Management

The study used a web-based research system for data collection and management. The project was housed within a research unit that has established experience in data collection using electronic devices. The use of this approach allowed for quality control to be implemented as data was being collected, which, in turn, improved the accuracy of the information collected. Data collected from the field were automatically transferred via a secured internet connection to the central database, which was maintained on an on-going basis. Biological samples were collected and stored in bar-coded containers, which facilitated the incorporation of laboratory results onto the main database.

### 2.7. Statistical Analysis

The required data were downloaded from the electronic database. A database was then created and cleaned in Excel. While in Microsoft Excel 2010 (Excel 365 for Windows, Redmond, Washington D.C., USA), some data (responses) were recoded and/or collapsed for more meaningful analysis. Data coherence, validity, reliability, and exploitability were checked. Descriptive data analysis was done using IBM SPSS Statistics version 25 (IBM, Armonk, New York, NY, USA). At the first level of analysis, univariate analysis or frequencies were run on all variables in the questionnaires. For descriptive purposes, frequencies were tallied, and percentages were calculated. At the second level of analysis, cross-tabulations were conducted to establish differences between groups. Pearson-product moment correlation coefficients were used to measure the strength of linear associations between two variables. Results were expressed in means and standard deviations for continuous variables and counts and percentages for categorical variables.

### 2.8. Bio-Analysis

Biochemical analyses were conducted in PathCare laboratories. Plasma glucose levels were measured by enzymatic hexokinase method (Beckman AU, Beckman Coulter, Cape Town, South Africa). Glycated haemoglobin (HbA1c) was analyzed with high-performance liquid chromatography (Biorad Variant Turbo, BioRad, Johannesburg, South Africa).

Ethical clearance was obtained from the ethics committee of the South African medical research council (SAMRC) (approval no. EC018-7/2015).

## 3. Results

### 3.1. Community Screening

The random sampling technique proved to be ineffective, with less than 10% of those selected through this approach consenting to be considered for the study. The self-selection process yielded a more positive response. A thousand and one (1001) adults were screened in eight areas (four black and four mixed-ancestry townships) during this pilot study, of which 158 individuals (59.5% mixed-ancestry, 76% female, mean age = 59.7 years (11.4 SD)) had already been diagnosed with T2DM (Figure 1).

Of the remaining, *n* = 843 (53% black, 77% female, mean age 47.3 (10.6 SD)), screened for T2DM and prediabetes, 74% had a BMI ≥ 25kg/m^2^, 24% had hypertension (BP ≥ 140/90 mmHg or known hypertension), and 43.4% (*n* = 354) were identified as at high risk of T2DM and prediabetes (Table 4). Three hundred and sixteen (89.3%) at-risk individuals presented themselves at the clinic for further investigation (81% female, 55% black, mean age = 51.8 years (9 SD)) (Figure 1).

### 3.2. Baseline Evaluation (Clinic Screening)

#### Socio-Demography

In the overall sample (*N* = 316), there was 54.4% black, 80.1% female, with a mean age of 51.8 years (SD = 8.9) (Table 5), and 43% had a high school (grade 8–11), and 27.2% had grade 12 and higher. Of the study sample, 38.9% were unemployed, 23.8% received a grant (pension/disability/child), and the majority (75.6%) had a monthly household income of less than R3201 (±218 $) (Table 5). There were differences between the population groups of mixed ancestry vs. black participants who were significantly older (mean age: 54 (7.7) vs. 49 (9.5)), less educated (tertiary education: 9% vs. 22.6%), and less unemployed (32.8% vs. 49.1%). However, employment levels were comparable between the groups, while a significantly higher proportion of mixed-ancestry participants were fulltime homemakers. Further, mixed-ancestry participants had higher income levels, with 33.7% of household income above R6401 p/m vs. 13.7% for black participants.

### 3.3. Health Risks

In this high-risk population, mean BMI and waist circumference (WC) were 36 (7.7) and 104 (13.2) (Table 6), respectively, with no significant differences between population groups. Only 3.8% of the sample had a normal BMI, 21.2% was overweight, and 73.7% was obese. The prevalence of hypertension was 30%, HbA1c > 5.7% was 68%, impaired fasting glucose (IFG) was 5.8%, and impaired glucose tolerance (IGT) was 13.2%. Diabetes was diagnosed in 10% of the sample. Forty five percent of the population reported having at least one family member with T2DM.

A significant difference was noted between the black and mixed-ancestry populations in the occurrence of elevated HbA1c (*p* < 0.05).

The prevalence of dyslipidemia was as follows: total cholesterol ≥ 5 mmol/L: 48%, high-density lipoprotein cholesterol (HDL-C) < 1.2 mmol/L: 52.9%, low-density lipoprotein cholesterol (LDL-C) ≥ 3 mmol/L: 57.7%, and triglycerides (TG) > 1.5mmol/L: 35.8%. Mixed-ancestry participants compared with their black counterparts had significantly worse profiles for all lipid parameters.

Regarding alcohol consumption, 13.3% of participants drank several times per week, while 55.1% abstained. Over a quarter (26.3%) of participants were current smokers. By population group, the prevalence of alcohol abstainers was significantly higher in the mixed ancestry vs. black group (45.3% vs. 65.8%). The former population compared with the latter was more likely to be current smokers (20.9% vs. 49%) (Table 6).

When comparing normoglycemia, dysglycemia (participants with impaired IFG and/or IGT), and diabetic groups, the overall cardiovascular disease (CVD) risk profile did not vary significantly except for HbA1c, with 100% of the diabetic group having raised HbA1c levels, compared to 78% (dysglycemia) and 59.4% (normoglycemia) (Table 7).

## 4. Discussion

The low uptake of the door to door home-based screening prevented us from capturing a random sample. We suspect that the high violence and crime rates, including property-related crime, in Cape Town was a major contributing factor in the poor uptake of home-based screening [34]. The self-selection method proved to be more practicable, with over 1000 participants arriving to be screened at community-based venues over eight months (August 2017–March 2018). Accordingly, learnings from the original US diabetes prevention program advocated the usefulness of using a range of recruitment strategies and that the most successful methods may differ depending on variables, such as age, gender, and race/ethnicity [35].

Of those who were identified as at risk of developing diabetes, close to 90% (316 out of 354) presented themselves for confirmatory biochemical testing at our research clinic.

Females made up the majority (80%) of the participants. This could be indicative of the differences in health-seeking behaviors within gender, which has been noted in the literature. However, health-seeking behaviors are complex, and gender is only one of the many contributors; other factors that have been highlighted are age, education, and socio-economic status [36,37]. In South Africa, specifically, people in urban areas have rated T2DM as having a larger impact on livelihood than those in rural areas [37]. Moreover, in South Africa, females do experience a higher burden of diabetes than their male counterparts (11.8% vs. 7.7%) [38]. Females also experience higher overweight (62.2% vs. 41%), obesity (36% vs. 14.6%), and physical inactivity (53.1% vs. 40.5%) rates compared to males, putting females at greater risk for non-communicable diseases overall [38].

Among the participants who presented at the clinic, 67.9% had elevated HbA1c levels, including 13% with dysglycemia, classifying them as prediabetic, and 10% with newly diagnosed diabetes. This equated to close to 70% of the sample identified by the ADRS as high-risk as having prediabetes or undiagnosed T2DM.

This highlights the need for community-based risk screening, followed by confirmatory blood tests for those identified at risk. The early identification of screen-detected diabetes, followed by optimal care, may prevent or delay debilitating diabetes-related morbidity and mortality [39,40]. Identifying people with prediabetes creates an opportunity for prevention or, at the very least, to delay the onset of T2DM through lifestyle interventions [8,9].

Furthermore, the high cardiovascular disease (CVD) risk factor (i.e., obesity, dyslipidemia, and hypertension) burden among participants identified with high-risk on ADRS but with normoglycemia on biochemical analysis support the view that once ranked as high-risk for diabetes, people will benefit from lifestyle interventions, either for diabetes risk or for general CVD risk reduction. Indeed, Sattar (2013) [41] concluded that glucose-based measures might not improve CVD risk prediction in those without diabetes. Therefore, diabetes risk screening/reduction cannot be considered in isolation from the overall CVD risk screening/reduction strategy.

As part of the SA-DPP lifestyle intervention, we developed culturally and socio-economically appropriate intervention material that addressed the barriers to optimal nutrition and physical activity to promote healthy lifestyle behaviors in South African communities using evidence-based and participatory approaches [25]. This lifestyle intervention, largely based on evidence from previous and ongoing diabetes prevention programs [8,9,10], hopes to reduce the T2DM and CVD risk of those identified at definitive future risk for T2DM.

A systematic review and meta-analysis have found that lifestyle interventions result in significant improvements in CVD risk (such as systolic BP, diastolic BP, TC, LDL-C, HDL-C, and TG), irrespective of glycemic or diabetes status [42].

### Strengths and Limitations

A strength of this study was that it was adaptive in nature and that early learnings inherently led to the successful recruitment of study participants without compromising the scope of our research. This learning in community screening efforts is key for future research efforts and others doing research in South Africa, the greater Africa, and other low-income countries. A limitation of this study was that the sample was small (pilot phase of the SA-DPP) and restricted to the Cape Town Metro (inherently different from other Metros) in the Western Cape of South Africa, and as such findings on CVD risk should be interpreted with caution.

## 5. Conclusions

Evidence has established the benefits of early detection of unknown diabetes and prediabetes for the individual (quality of life), as well as the burden (cost) on the health system [1]. Community-based screening efforts in low-resourced settings, such as South Africa, seems to be a feasible method for early detection, as corroborated by our pilot phase findings. Cost-effectiveness needs to be determined in this setting, but effectiveness has been established [6].

## Figures and Tables

**Table 1:** Area demographics of included communities.

Area	Total Population	Level of Education	Average Income	Number of Schools	Number of Health Facilities
Khayelitsha SP (informal settlement) *Predominantly black community*	11,251 (2357.23/km^2^)	No info	No info	No info	No info
Belhar (Ward 12 and 22) *Predominantly mixed-ancestry community*	56,234 (6882.25/km^2^)	No schooling aged 20 + (1.4%). Higher education aged 20 + (9.4%). Matric aged 20 + (29%).	Average household income: No income = 8.3%. R1–R4800 = 1%. R4801–9600 = 1.8%. R9601–R19,600 = 8.9%. R19,601–38,200 = 14.9%. R38,201–76,400 = 20.2%. R76,401–R153,800 = 20.8%. R153,801–R307,600 = 15.3%. R307,601–R614,400 = 6.9%. R614,401–R1,228,800 = 1.4%.	±20	2 (St Vincent clinic and Chestnut CHC)
Athlone (Ward 49) *Predominantly mixed-ancestry community*	8893 (5900/km^2^)	71.9% completed grade 9 or higher. 35.6% completed matric or higher.	Average household income: No income = 11.5%. R1–R4800 = 1.7%. R4801–9600 = 3.3%. R9601–R19,600 = 15.5%. R19,601–38,200 = 18.6%. R38,201–76,400 = 18.5%. R76,401–R153,800 = 15%. R153,801–R307,600 = 9.6%. R307,601–R614,400 = 4.7%. R614,401–R1,228,800 = 1.2% .Average annual income = R57,500	±20	6 (Hood road medical center, Dr. Abdurahman day hospital, Samwumed, Fresenius medical care and Athlone kidney and Dialysis center, Al-nisa maternity home, and Kromboom dental center)
Bongweni *Predominantly black community*	1791 (9420.58 per km^2^)	No info	No info	±11 to 15	3 (Khayelitsha community health clinic, Clinimed medical and asthetic solutions, and mens clinic international)
Lavender hill (Ward 68) *Predominantly mixed-ancestry community*	26,372 (9335.6/km^2^)	62.3% completed grade 9 or higher. 26.7% completed matric or higher.	Average annual income = R57,500	±7	2 (Lavender hill clinic and Sea wind CHC–TB unit)
Gugulethu (formal and informal housing) (Ward 40 and 41) *Predominantly black community*	98,468 (15,161.70/km^2^)	78.2% completed grade 9 or higher. 44.2% completed matric or higher.	Average household income: No income = 19.3%. R1–R4800 = 5.3%. R4801–9600 = 7.1%. R9601–R19,600 = 16.5%. R19,601–38,200 = 23.2%. R38,201–76,400 = 15.4%. R76,401–R153,800 = 8.5%. R153,801–R307,600 = 3.4%. R307,601–R614,400 = 1.1%	±20	2 (Gugulethu medical center and KTC Gugulethu CHC)
DuNoon (formal and informal housing) (ward 104) *Predominantly black community*	29,268 (29,518.5/km^2^)	70.7% completed grade 9 or higher. 27% completed matric or higher.	Average monthly income = R2400 Average annual income = R30,000	5	1 (DuNoon CHC)
Lotus river (ward 65) *Predominantly mixed-ancestry community*	38,143 (7615.72/km^2^)	74.8% completed grade 9 or higher. 40.9% completed matric or higher.	Average annual income = R57,500	±10–15	2 (Lotus river community health clinic and lotus river public clinic)

Sources: Total population data—Census2011.adrianfrith.com [14]; Level of education data for Athlone, Lavender hill, Gugulethu, Dunoon, Lotus river—WaziMap.co.za [15]; Level of education for Belhar—Statssa.gov.za [16]; Average household income data for Belhar, Athlone, Gugulethu—Statssa.gov.za; Average annual income data for lotus river, Lavender hill, Dunoon—WaziMap.co.za [15]; Number of schools and number of health facilities data—Google maps [17]. CHC—community health center; TB—tuberculosis.

**Table 2:** The African diabetes risk score, in those without prior diabetes. SADPP, South African diabetes prevention program.

SADPP Risk Score Calculation	
Regression coefficients of the model	
Variable	Coefficient
Age (per 1-year increase)	0.045
Waist Circumference (per cm increase)	0.048
Hypertension (present (1) vs. absent (0))	0.649
Intercept	−11.012

Diabetes score = 100 × (1 ÷ (1 + exp (−(−(11.012) + 0.045 × A+0.048 × ((B + C) ÷ 2) + 0.649 × (D))))). Where A–Age, B–Waist circumference 1 in cm, C–Waist circumference 2 in cm, D–Hypertension: Yes, if Systolic = 140 or more or Diastolic = 90 or more or if self-reported history of doctor diagnosed hypertension. Participants with a score >1.46 (Black) or >1.15 (mixed ancestry).

**Table 3:** Measurement domains, tools, and data collection for the South African Diabetes Prevention Programme (SADPP) pilot phase.

Variable	Components	Measurements Tools/Questions	Reporting in this Paper
**General information**		Personal details and contacts for follow up	Not reported
**Socio-demographic measures**		Age, gender, area, community, current marital status, education level, employment, income	Reported
**Self-reported medical history**	General		Not reported
	Chronic diseases	Diabetes, hypertension, cholesterol, bronchitis/chronic obstructive pulmonary disease, cancer, tuberculosis	Not reported
	Heart health	Jackson heart medical form [24]	Not reported
	medication	Chronic prescription medication	Not reported
	Family medical history	Hypertension, diabetes, heart attack, stroke, cancer	Only familial diabetes history reported
**Behavioral measures**	Tobacco use	WHO STEPS questionnaire [22]	Reported
	Alcohol use	WHO STEPS questionnaire [22]	Reported
	Sedentary behavior	Time spent in front of a screen	Reported elsewhere [25]
	Sleep	Time, quality	Reported elsewhere [25]
**Dietary Measures**	24-hour dietary recall Barriers to healthy eating	Single unquantified dietary recall [26]; food frequency for processed food, barriers to fruit and vegetable consumption [27]	Not reported
**Physical activity measures**	Physical activity pattern	WHO STEPS questionnaire: global physical activity questionnaire (GPAQ) [22]	Reported elsewhere [25]
	Barriers to physical activity	Scale adapted from the one designed by Booth et al. [22]	Reported elsewhere [25]
	Self-efficacy	Scale adapted from the exercise self-efficacy scale (ESES) designed by Schwarzer and Jerusalem [28]	Reported elsewhere [25]
**Clinical measures**	Waist circumference	Measured between the lower border of the lowest rib and upper border of the iliac crest/pelvic bone to the nearest 0.1 cm	Reported
	Weight	Weight measurement with minimal clothing on a digital (SECA) scale, recorded to the nearest 0.1 kg	Reported (BMI)
	Height	Standing height, minimal clothing, aligning head in a standard anatomical position using a SECA stadiometer	
	SBP	Electronic M6 COMFORT OMRON device with an integrated cuff	Reported
	DBP HbA1c	Electronic M6 COMFORT OMRON device with an integrated cuff HbA1c measured using fasting blood and HPLC	Reported
**Neighborhood indicators**	Stores and facilities, access to services and places, roads and walking paths, places for walking/cycling/playing, surroundings, safety from crime and traffic, personal safety, stranger danger	Neighborhood environment walkability scale (NEWS) Africa Questionnaire [23]	Reported elsewhere [25]
**Psychological measures**	Chronic stress	Chronic stress scale [29]	Reported elsewhere [25]
	Mood (depression and anxiety)	Patient health questionnaire-9 amended in line with CURES-65 study [30], general anxiety disorder scale [31]	Not reported
	Support networks	ENRICHD social support scale [32]	Not reported
	Quality of life	The MOS 36-item short-form health survey [33]	Not reported
	Life satisfaction	How satisfied are you with your life as a whole?	Not reported

Adapted from Hill et al. 2020 [21]. WHO—World Health organization; BMI—body mass index; SBP—systolic blood pressure; DBP—diastolic blood pressure; HbA1c—glycated haemoglobin; HPLC—high-performance liquid chromatographic.

**Table 4:** Characteristics of community screened participants.

Characteristic		*N*	%
Ethnicity	Black	432	52.9
	Mixed ancestry	385	47.1
Sex	Males	189	23
	Females	648	77
Mean age (years)	47.3 (10.6 SD)		
Older than 45	Yes	512	61.2
Township	Athlone **	85	10.4
	Belhar **	145	17.4
	Bongweni/Tembani *	19	2.4
	Du Noon *	49	5.7
	Gugulethu *	239	28.3
	Khayelisha SP *	133	15.7
	Knole park/Lotus river **	75	8.9
	Lavender hill **	86	10.2
Body mass index	Underweight	27	3.3
	Normal weight	181	22.3
	Overweight	208	25.6
	Obese	396	48.8
Mean waist circumference (cm)	94 (21.9 SD)		
BP ≥ 140/90 mmHg or known hypertension	Yes	198	24.2
Family history of diabetes	Yes	244	29.2
At risk for diabetes	Yes	354	43.4

* Black; ** Mixed ancestry.

**Table 5:** Socio-demographic characteristics of participants who presented for clinic screening.

Socio-Demographic Characteristics (*N* = 316)				
		General Population	Black 177 (54.2%)	Mixed-Ancestry 141 (45.7%)
Age, mean (SD)	51.8 (8.9)		49 (9.5)	54 (7.7) **
Gender	*N*	%	*N* (%)	
Male	60	19.0	30 (17.5)	30 (20.8)
Female	253	80.1	141 (82.5)	114 (79.5)
NA	3	0.9		
Education				
Never went to school	2	0.6	1 (0.6)	1 (0.7)
Primary school (grades 1–7)	76	24.1	36 (21.4)	41 (28.3)
High school (grades 8–12)	136	43.0	67 (39.9)	69 (47.6) *
Less than grade 12 + FET*/certificate/diploma	11	3.5	6 (36.)	5 (3.4)
Grade 12	36	11.5	20 (11.9)	16 (11)
Tertiary/diploma/degree	51	16.3	38 (22.6)	13 (9) *
Not Assigned	5	1.6		
Occupation				
Employed (full- or part-time/self-employed)	93	29.4	53 (29.9)	40 (28.6)
Unemployed	133	42	87 (49.1)	46 (32.8)
Full-time homemaker	21	6.6	2 (1.1)	19 (13.5)
Pensioner	58	18.4	21 (11.9)	37 (26.2)
On a disability grant	13	4.1	5	8
Child grant	4	1.3	3	1
Income				
No income	32	10.2	23 (13.7)	9 (6.2) **
R1–R400	12	3.8	12 (7.1)	0
R401–R800	27	8.6	18 (10.7)	9 (8.6)
R801–R1600	75	24	41 (24.4)	34 (23.4) **
R1601–R3200	95	30.4	51 (30.4	44 (30.3)
R3201–R6400	42	13.2	16 (9.5)	26 (17.9) **
R6401–R12,800	19	6.1	4 (2.4)	15 (10.3) **
R12,801–R25,600	10	3.2	3 (1.8)	7(4.8) **
R25,601–R51,200	1	0.3	0	1 (0.7)

FET*–Further education and training. * *p*-value < 0.5; ** *p*-value ≤ 0.001.

**Table 6:** Health and behavioral risk factors among participants who presented for clinic screening.

Risks Factors (*N* = 316)				
	General Population		Black	Mixed-Ancestry
Body Mass Index (BMI, kg/m^2^), mean (SD)	36 (7.7)		36.9 (8.3)	35.1 (6.9)
Waist circumference (cm), mean (SD)	104 (13.2)		103.9 (13.9)	104.4 (12.5)
BMI	*N*	%	*N* (%)	*N* (%)
Underweight (<18.5)	X0	0	0	0
Normal weight (18.5–24.9)	12	3.8	(4.1)	(3.6)
Overweight (25.0 to 29.9)	67	21.2	(21.6)	(20.1)
Obese	233	73.7	(74.3)	(76.3)
Not documented	4	1.3		
Family medical history				
Having at least one known diabetic close relative	141	44.6	70 (40.9)	71 (49.7)
Don’t have one known diabetic close relative	57	18.0	99 (57.9)	68 (47.6)
Don’t know	114	36.1	2 (1.2)	4 (2.8)
Not documented	4	1.3		
Blood pressure				
Optimal/normal (<120/120–129 mmHg/<80/80–84 mmHg)	129	40.8	(35.7)	(47.5) *
High normal (130–139 mmHg/85–89 mmHg)	71	22.5	(25.7)	(19.4) *
Hypertension (≥140 mmHg/90 mmHg)	93	29.4	(33.3)	(25.9) *
Isolated systolic hypertension (≥140 mmHg/<90 mmHg)	19	6.0	(5.3)	(7.2) *
Not documented	4	1.3		
Glycosylated hemoglobin (HbA1c)				
≥5.7 mmol/L	208	67.9	104 (62.3)	104 (74.3) *
Not documented	11	3.5		
Glycemia				
Impaired fasting glucose (>6.1–7 mmol/L)	18	5.8	10 (5.9)	8 (5.8)
Impaired glucose tolerance (≥7.8–11.1 mmol/L)	41	13.2	22 (12.9)	19 (13.7)
Diabetic (IFG >7 mmol/L and IGT >11.1 mmol/L)	31	10	15 (8.8)	16 (11.3)
Cholesterol				
Total cholesterol (>5 mmol/L)	150	48.2	68 (40)	82 (58.2) **
HDL cholesterol (<1.2 mmol/L)	166	52.9	79 (47)	85 (60.3) *
LDL cholesterol (>3 mmol/L)	179	57.7	78 (47.6)	99 (70.2) **
Triglycerides (>1.5 mmol/L)	113	35.8	47 (27.6)	63 (44.7) **
Alcohol consumption				
Abstainer	174	54.8	78 (45.3)	98 (65.8) **
Less than once a month	51	15.9	32 (18.6)	19 (12.8) **
1–3 days per month	45	14	30 (17.4)	15 (10.1) **
Several times per week	42	13.1	31 (18.0)	11 (7.4) **
Not documented	7	2.2		
Tobacco status				
Non-smoker	191	59.95	125 (72.7)	66 (44.3) **
Current smoker (daily or occasionally)	109	34	36 (20.9)	73 (49) **
Ex-smoker	14	4.4	10 (5.8)	4 (2.7) **
Not documented	7	2.2		

* *p*-value < 0.5; ** *p*-value ≤ 0.001; cm—centimeter; IFG—impaired fasting glucose; IGT—impaired glucose tolerance; HDL—high-density lipoprotein; LDL—low-density lipoprotein.

**Table 7:** Significant differences in cardiovascular disease (CVD) risk factors and glycemic status.

Glycemic Status	Normoglycemia (*n* = 239)	Dysglycemia (*n* = 41)	Diabetic (*n* = 31)	*p*-Value
	(IFG < 6 and IGT < 7.8)	(IFG > 6.1–7 mmol/L and IGT ≥ 7.8–11.1 mmol/L)	(IFG > 7 mmol/L and IGT > 11.1 mmol/L)	
HbA1c (≥5.7 mmol/L)	142 (59.4)	32 (78)	31 (100)	<0.0001
Total cholesterol (>5 mmol/L)	116 (48.5)	16 (39)	15 (46.8)	0.525
HDL cholesterol (<1.2 mmol/L)	84 (35)	19 (46.3)	15 (46.8)	0.159
LDL cholesterol (>3 mmol/L)	122 (51)	19 (46.3)	19 (59.4)	0.544
Triglycerides (>1.5 mmol/L)	77 (32.2)	16 (39)	15 (46.8)	0.169
BMI (overweight and obese) (>25 kg/m^2^)	229 (95.8)	39 (95.1)	30 (93.8)	0.484
Hypertension (≥140 mmHg/90 mmHg)	80 (33.5)	16 (39)	13 (40.6)	0.524

IFG—Impaired fasting glucose; IGT—impaired glucose tolerance; HDL—high-density; HDL—lipoprotein; LDL—low-density lipoprotein; BMI—body mass index.
